# Foetal magnetic resonance imaging: A necessity or adjunct? A modality comparison of *in-utero* ultrasound and ultrafast foetal magnetic resonance imaging

**DOI:** 10.4102/sajr.v25i1.2010

**Published:** 2021-03-19

**Authors:** Sunaina Ramdass, Sumaiya Adam, Zarina Lockhat, Andries Masenge, Farhana E. Suleman

**Affiliations:** 1 Department of Diagnostic Radiology, Faculty of Health Sciences, Steve Biko Academic Hospital, University of Pretoria, Pretoria, South Africa; 2 Department of Foeto-maternal Medicine, Faculty of Health Sciences, Steve Biko Academic Hospital, University of Pretoria, Pretoria, South Africa; 3 Department of Biostatistics, Faculty of Statistics, University of Pretoria, Pretoria, South Africa

**Keywords:** foetal MRI, ultrafast foetal MRI, *in-utero* ultrasound, antenatal sonar, comparison, congenital anomalies

## Abstract

**Background:**

Congenital anomalies occur in approximately 2% of newborns, resulting in severe medical, physical and social disabilities. Managing clinicians, therefore, require more confidence in their diagnosis and prognostic accuracy before appropriately counselling the parents regarding termination of pregnancy.

**Objective:**

The aim of this study was to investigate the role of magnetic resonance imaging (MRI) following the diagnosis of foetal anomalies at a foetomaternal unit of a tertiary South African institution.

**Methods:**

Eighty-eight pregnant women in their late second/third trimester who underwent both an ultrasound (US) at the foetomaternal unit and foetal MRI at the Radiology Department from 01 July 2013 to 30 September 2019 were included in this clinical study conducted at Steve Biko Academic Hospital.

**Results:**

Despite the high degree of concurrence (73.9%) between both modalities regarding the main diagnoses, MRI provided additional information in 45.5% of patients and changed the diagnosis in 25% of the patients. It further demonstrated superiority in providing diagnostic information in 97% of cases where the US alone was inadequate to counsel parents regarding the termination of pregnancy, and it completely changed the clinical management in 42% of cases.

**Conclusion:**

It is clearly evident from this study that foetal MRI is a necessity when termination of pregnancy is being considered following an US conducted by the foetomaternal unit. This allows for a complete foetal assessment and gives the managing clinician sufficient diagnostic confidence to prognosticate the future quality of life of the child.

## Introduction

Congenital anomalies are reported in approximately 2% of newborn children often because of an unknown aetiology, resulting in severe medical, physical and social disabilities.^1^ The *Choice of Termination of Pregnancy Act* (1996) of South Africa allows for termination of pregnancy after the 20th week, until term essentially, as agreed upon by two medical practitioners for major structural anomalies that would result in poor quality of life.^2^ Managing clinicians, therefore, require more diagnostic confidence and prognostic accuracy on the anomaly or complex anomalies present before appropriately counselling the parents.^1,3^

Ultrasound (US) is the current global method for screening congenital anomalies, which has many limitations. Ultrafast foetal magnetic resonance imaging (MRI) could potentially play a significant role as a new modality in comprehensively diagnosing and prognosticating congenital abnormalities detected at US. Considering the cost, waiting lists and personnel required for an MRI, we need to be certain that it adds considerable value to the outcome of the pregnancy.

Ultrasound is relatively inexpensive, easily accessible and reliant on a single-trained sonographer. The development of new technology in US has been revolutionary in the past decade, allowing for even four-dimensional *in-utero* assessment. There is, however, very little consensus as to the sensitivity as well as the specificity of US, with studies showing sensitivity ranging from 14% to 92% for major structural abnormalities within the second trimester.^4,5^ Ultrasound is a safe, real-time imaging technique that provides assessment of foetal and placental health.^1^ However, it is dependent on the operator and the foetal position, and is of little value in obese mothers and in oligohydramnios.^1,6^ Transvaginal sonography has a role to play in obese patients, early pregnancy dating and first trimester bleeding. Neurosonography is performed by specially trained individuals at tertiary centres depending on the gestational age at the time of referral.

Foetal MRI is a highly specialised investigation, which was not initially used in obstetrics because of the long acquisition times. However, with the advancement of ultrafast imaging, MRI allows for three-dimensional imaging of the whole foetus *in-utero* with extremely short acquisition times.^1^ Magnetic resonance imaging is an extremely expensive study, which also requires trained radiographers for acquisition and radiologists for interpretation. It is a very limited resource, especially within our centre. Foetal MRIs are usually conducted after 18 weeks of gestation as certain organ systems are only fully developed after this gestational age, and the foetus is large enough to allow visualisation of almost all of the anomalies present. The corpus callosum, for example, is a common indication for MRI referral and is only fully formed after 20 weeks. At this gestation, foetal motion is also less of a factor.

Vimercati et al.^5^ correlated the US diagnoses and autopsy findings over 7 years. They observed that in 49% of cases, US and autopsy findings were in complete agreement with one another. An additional 22% of anomalies that occurred were not detected on US, and in 4% of cases there was a complete lack of agreement. Of significance, they also noted that the degree of agreement was higher when ultrasounds were performed at tertiary centres.

There are many published studies on the comparison of these modalities for neurological anomalies, and in particular the brain.^1,3,7,8,9^ Fewer studies assess all the organ systems, and fewer studies still look at focused non-neurological systems. They all agree that for posterior fossa assessment, MRI is superior. It was determined in specialised units that the combination of neurosonography and foetal MRI enables accurate diagnosis of most posterior fossa anomalies.^10^

A study assessing the contribution of MRI to foetal US by Kul et al.^11^ determined that US was superior in 4% of cases and MRI in 39% of cases. Another comparative study over a 10-year period observed that MRI changed the findings of foetal US, resulting in a change of parental counselling in 68.4% of cases.^12^

The overall consensus, however, is that MRI is best viewed as an adjunct to US.^1,4,6,8,12,13,14^ It is also clear that even just the confirmation of the US findings by MRI and the exclusion of other anomalies should be viewed as clinically relevant as it assists clinicians with the counselling of parents.

The results vary in other studies; consensus between US and MRI was observed in 13% – 50% of cases, MRI was found to provide additional information in 17% – 100% of cases and change the diagnosis in 11.9% – 100% of cases.^1,4,7,8,9,13,14,15^ It is also clear that MRI proves to be superior in assessment of imaging and delineating pathologies of the thorax, abdomen and urogenital systems.^4,16,17^ A recent study observed an increase from 2.9% to 5.1% in parental request for termination of pregnancy following an MRI, which confirmed and/or provided additional information.^18^ A single South African study noted in the literature, comparing US to MRI and postnatal outcomes noted that the commonest reason for referral to MRI at their centre was ventriculomegaly, with good agreement between foetal US and MRI but poor agreement with postnatal findings due to the resolution of the ventriculomegaly.^19^

Given the varying statistics and the importance of diagnostic accuracy and prognostication, it was prudent to assess the role of foetal MRI in comparison with prenatal *in-utero* ultrasonography at a local tertiary institute, Steve Biko Academic Hospital.

## Materials and methods

Eighty-eight pregnant women in their late second or third trimester who underwent both an US at the foetomaternal unit and a foetal MRI at Steve Biko Academic Hospital Radiology Department from 01 July 2013 to 30 September 2019 were included in this clinical study. Patients were only referred for a foetal MRI scan when the foetomaternal specialists came to a conclusion that US was inadequate for formulating a decision.

The foetomaternal unit used a Phillips Affiniti 70 sonar machine with an abdominal probe or transvaginal probe. The patients were examined in the supine or left lateral position. The foetomaternal specialists and fellows in training performed the US examinations. Whilst the US were conducted by various people during the study period, all US were reviewed by one or more of the three foetomaternal specialists in the department. Patients were then referred for MRI and scans were performed within the late second and third trimester. A 1.5T Philips MRI scanner with a body coil was used for all the foetal MRIs performed at our facility. No sedation was provided to the mother. All patients were scanned in a supine position, and sequences were obtained in a three-dimensional plane. Sequences included Balanced Fast Field Echo (time of repetition 3.8 and time to Echo 1.9) and Single-shot Turbo Spin Echo (time of repetition 15 000 and time to Echo 120) in three planes. The slice thickness used was 4 mm. The total acquisition time was usually 10–15 min. Patients were subsequently followed up at the foetomaternal unit, counselled regarding the MRI findings and advised on termination of pregnancy where appropriate.

A direct comparison was made between the findings at US and MRI. The data analysis included frequency tables (counts and percentages), performed to describe the MRI and US diagnoses and inferential statistics using the McNemar (two sample) and Cochran’s (K sample) test to measure whether the combinations of categorical values between MRI and US were equally likely.

### Ethical considerations

This protocol was approved by the Ethics committee of a Gauteng University (Reference No. 547/2019). As this was a retrospective study, the ethics committee agreed to waive patients’ consent.

## Results

A total of 88 patients underwent a prenatal *in-utero* US and an MRI from 01 July 2013 to 30 September 2019. The maternal age ranged from 16 to 43 years with an average age of 29 years, and the gestational age ranged between 17 to 41 weeks with an average gestational age of 28 weeks at the time of referral for MRI.

Ultrasound and MRI findings were in concordance in 73.9% of cases and were in discordance in 25% of cases (Table 1). Only one of the MRIs was inconclusive because of rapid foetal movement. The MRI scans confirmed the US findings in 26.1% of patients and provided additional information in 45.5% of patients (Table 2). The findings at MRI changed the diagnosis in 27.3% of patients. In 16.7% of these cases, MRI findings changed the diagnosis to normal, whereas US detected 24 false positives and 7 false negatives (Table 3).

**TABLE 1:** Frequency and percentage agreement between ultrasound and magnetic resonance imaging.

Variable	Frequency	*%*
No agreement	22	25.0
Unknown or inadequate study	1	1.1
Agreement	65	73.9
**Total**	**88**	**100.0**

US, ultrasound; MRI, magnetic resonance imaging.

**TABLE 2:** Magnetic resonance imaging contribution.

Variable	Frequency	*%*
MRI confirmed US diagnosis	23	26.1
MRI provided additional information	40	45.5
MRI changed the diagnosis	24	27.3
Unknown	1	1.1
**Total**	**88**	**100.0**

US, ultrasound; MRI, magnetic resonance imaging.

**TABLE 3:** Abnormalities on ultrasound when compared with magnetic resonance imaging.

Modality	Count	MRI		Total
		No abnormality detected	Abnormality detected	
US	No abnormality detected	480	7	487
	Abnormality detected	24	104	128
	**Total**	**504**	**111**	**615**

US, ultrasound; MRI, magnetic resonance imaging.

Following a review of the MRI findings (Table 4), 36.4% of parents were counselled in favour of termination of pregnancy. In 22.7% of cases, parents were counselled not to terminate the pregnancy, and 38.6% are unknown.

**TABLE 4:** Frequency and percentage of termination of pregnancy.

Variable	Frequency	*%*
Intrauterine death	2	2.3
No termination advised	20	22.7
Unknown	34	38.6
Termination advised	32	36.4

**Total**	**88**	**100.0**

Upon retrospective analysis, MRI changed the decision for proceeding with termination of pregnancy in 25% of patients where US had initially advised against termination. One such example included a patient referred for assessment of foetal ventriculomegaly noted on the US. The findings at MRI revealed alobar holoprosencephaly, and hence, termination was advised. In 13.8% of patients, MRI changed the decision against proceeding with termination, where US had initially advised for termination (Table 5). One such example included a patient referred for possible Dandy Walker malformation, corpus callosal agenesis and ventriculomegaly on the US. Magnetic resonance imaging noted prominent ventricles with no other associated abnormalities. Overall MRI allowed clinicians to counsel parents regarding the termination of pregnancy in 97% of cases where US was insufficient. The findings at MRI had a direct impact on the clinical management in 42% of cases either by providing the complete diagnostic picture to allow a decision to be made or by changing the initial decision.

**TABLE 5:** Late termination of pregnancy ultrasound compared to magnetic resonance imaging.

Late TOP	Variable	Late TOP MRI			Total	Measure of agreement	
		More information	No	Yes		Kappa	*p*
US	More information	1	16	13	30	0.229	0.000
	No	0	3	1	4	-	-
	Yes	1	7	46	54	-	-
**Total**	**-**	**2**	**26**	**60**	**88**	**-**	**-**

US, ultrasound; MRI, magnetic resonance imaging; TOP, Termination of pregnancy.

Cohen’s Kappa score is a measure of inter-rater reliability. This is a robust measure as Kappa takes into account the possibility of the agreement occurring by chance. The overall Cohen’s Kappa score between US and MRI regarding the late-term termination of pregnancy was fair (kappa = 0.229). The central nervous system, thoracic, abdominal and gestational systems showed a Cohen’s Kappa score that was substantial (Table 6). Whilst the spinal and urogenital systems indicated the highest score of almost perfect, the musculoskeletal system revealed the lowest score of moderate agreement.

**TABLE 6:** Cohen’s Kappa scores per organ system.

US	Variable	MRI		Total	Measure of agreement	
		No abnormality detected	Abnormality detected		Kappa	*p*
Central nervous system	No abnormality detected	14	1	15	-	-
	Abnormality detected	7	65	72	-	-
	Total	21	66	87	0.722	0.000
Spinal	No abnormality detected	79	0	79	-	-
	Abnormality detected	1	8	9	-	-
	Total	80	8	88	0.935	0.000
Thoracic	No abnormality detected	69	2	71	-	-
	Abnormality detected	6	11	17	-	-
	Total	75	13	88	0.68	0.000
Abdominal	No abnormality detected	80	2	82	-	-
	Abnormality detected	1	5	6	-	-
	Total	81	7	88	0.751	0.000
Urogenital	No abnormality detected	79	0	79	-	-
	Abnormality detected	2	7	9	-	-
	Total	81	7	88	0.863	0.000
Musculoskeletal	No abnormality detected	77	2	79	-	-
	Abnormality detected	4	5	9	-	-
	Total	81	7	88	0.588	0.000
Gestational	No abnormality detected	82	0	82	-	-
	Abnormality detected	3	3	6	-	-
	Total	85	3	88	0.651	0.000

US, ultrasound; MRI, magnetic resonance imaging.

## Discussion

The gestational age at the time of the scan is of importance, as there is less foetal movement and better delineation of anatomy in the older foetus. There is, therefore, consensus that MRI must be performed in or after the second trimester. The mean gestational age of 28.13 weeks in this study is within the range of the other studies, which allows for comparative assessments. The mean gestational age in the literature ranged from 24.8 to 32.2 weeks.^7,11,13,15^ It can be observed from the study by Di Masicio et al.^18^ that a difference in the rate of central nervous system abnormalities was not detected clinically at birth when foetal MRI was carried out before, compared with after, 24 weeks of gestation.

There was a much higher percentage of agreement between US and MRI in this study (73.9%) than that noted in the reviewed literature (28.6% – 52.0%).^4,9,12^ There was a much lower percentage of disagreement of 25% between modalities when compared with the varying statistics of other studies ranging from 35.0% to 47.6%.^4,9,11^ US is an operator-dependent modality, and the foetomaternal specialists and fellows are all highly trained and specialised. These outcomes may reflect a high standard of sonography performed at Steve Biko Academic Hospital’s tertiary unit.

The findings of MRI confirmed the US findings in 26.1% of patients and provided additional information in 45.5% of patients, which is a significant percentage, when one considers the importance of a clinician needing to be as well informed as possible to accurately prognosticate the quality of life and counsel parents regarding the termination of pregnancy. Carbone et al.^12^ evaluated 328 patients over a period of 10 years and found that MRI changed the diagnosis in 68.4% of patients, compared with just 27.3% in this study, and altered that diagnosis to normal in 11.9% of patients, compared with 16.7% in this study.

In order to establish whether MRI changed the counselling of parents regarding the late-term termination of pregnancy, a senior foetomaternal specialist, who was blinded to the outcomes, reviewed the US and MR findings independently and noted whether she would have advised termination of pregnancy. Whilst many other studies mentioned the difficulty in being able to assess the clinical impact of MRI, it was observed in this study that MRI allowed clinicians to counsel parents regarding the termination of pregnancy in 97% of cases where US, as a standalone modality, proved inadequate. The MRI findings had an impact on clinical management in 42% of cases in this study, which was a lower percentage when compared with the 55% – 68.4%^5,14^ reported in the reviewed literature. This may be attributable to the higher degree of agreement between the modalities at our hospital.

Further assessments were made in evaluating the ability of both modalities in assessing anomalies of each organ system. The overall organ system reported a Cohen’s Kappa score of 0.839, which is almost perfect. This reflects a high degree of inter-rater reliability between the two modalities. This correlates with the overall agreement of 73.9% between US and MRI.

The central nervous system is the commonest system evaluated in the literature, as ventriculomegaly is the most frequent anomaly detected at sonography, and therefore, the usual indication for an MRI. Magnetic resonance imaging has well established its superior role in visualising the posterior fossa. Levine et al.^3^ showed that whilst US may also demonstrate ventriculomegaly, it fails to demonstrate the underlying aetiology, as well as associated abnormalities. An example of this includes when US demonstrates the characteristic massive ventriculomegaly and fails to demonstrate the cerebral aqueductal stenosis.

Ultrasound is poor at delineating the thorax as evidenced by the detection of six false positives and two false negatives in this study. Magnetic resonance imaging also proves to be superior in abdominal and urogenital assessment as it provides a much larger field of view, and therefore, a complete system assessment. Kul et al.^11^ revealed that additional information provided by MRI on thoracic and abdominal anomalies detected at US was 43.8% and 37.5%, respectively. The lowest score was noted in the musculoskeletal system, which demonstrated moderate agreement, probably because of the superior soft tissue delineation of MRI.

Ultrasound has the advantage of being real time and is able to assess placental health. It is also superior in assessing the vascularity of masses, and because of its cost effectiveness, allows for multiple follow-up studies to assess interval change. Magnetic resonance imaging is a three-dimensional evaluation that allows for the assessment of masses in the context of the foetus as a whole whilst assisting with surgical planning. As a result of the cost and personnel required, it cannot be used to assess interval changes.

It is important to note that foetal imaging is not available in many antenatal clinics across South Africa. As a result, many foetal abnormalities may be missed and patients are never referred for tertiary care. In addition, patients with discerned anomalies who are referred for assessment may fail to follow up under socio-economic circumstances, and this may account for the 38.6% of unknown outcomes in this study.

One of the limitations of this study was that patients were not followed up at postmortem or birth or in the subsequent neonatal period to review and compare the anomalies with those noted at US and MRI. This would have been an important criterion for assessment regarding the diagnostic accuracy of both these modalities. However, postmortems are not possible on all foetuses at delivery because of consent issues, macerated foetuses at delivery and resource constraints. A second limitation includes the technical system failure in the foetomaternal unit, which did not allow for access to older foetal US reports preceding July 2013, limiting the study population to a smaller number.

Future recommendations include the use of foetal (four dimensional [4D)] neurosonography, which may decrease the referral burden on MRI, the appointment of a dedicated radiologist interpreting all foetal MRIs, which will improve reporting at combined departmental discussions, and better post-mortem or birth follow-up (neonatal and childhood) to allow for the correlation of findings.

## Conclusion

It is clearly evident from this study, as in other studies from the literature, that foetal MRI is a necessity and not merely an adjunct, when termination of pregnancy is being considered following an US conducted by the foetomaternal unit. This allows for the most complete foetal assessment and allows the managing clinician to have enough diagnostic confidence to prognosticate the quality of life that the child will have.
